# Lifetime Mixed Depression and Childhood Trauma in Individuals With Bipolar Disorders

**DOI:** 10.1111/acps.70071

**Published:** 2026-01-25

**Authors:** Francesca Bardi, Mario Pinto, Alessio Simonetti, Marco Di Nicola, Marianna Mazza, Giovanni Manfredi, Georgios D. Kotzalidis, Gabriele Sani, Delfina Janiri

**Affiliations:** ^1^ Section of Psychiatry, Department of Neuroscience, Head‐Neck and Chest Università Cattolica del Sacro Cuore Rome Italy; ^2^ Section of Psychiatry, Department of Neuroscience, Head‐Neck and Chest Fondazione Policlinico Universitario Agostino Gemelli IRCCS Rome Italy; ^3^ Department of Psychiatry and Behavioral Sciences Baylor College of Medicine Houston Texas USA; ^4^ Centro Lucio Bini Rome Italy

**Keywords:** bipolar depression, bipolar disorder, childhood trauma, emotional trauma, mixed depression, physical trauma

## Abstract

**Background and Aims:**

Mixed Depression (MxD), characterized by the co‐occurrence of depressive and excitatory symptoms, is a prevalent yet often underdiagnosed presentation in bipolar disorders (BD), with significant implications for prognosis and treatment. Childhood trauma is a key environmental risk factor associated with a more severe course of BD, as it influences its onset, progression, and clinical presentation. To date, no studies have specifically investigated the relationship between lifetime childhood trauma and lifetime MxD in individuals with BD. The aim of our study was to address this gap in a large and well‐characterized sample of patients with BD type I and type II.

**Methods:**

A total of 376 individuals, 220 euthymic outpatients with BD (MxD = 100, non‐MxD = 120) and 156 healthy controls (HC), were enrolled. Childhood trauma was assessed using the Childhood Trauma Questionnaire (CTQ). Lifetime MxD was identified according to the Koukopoulos criteria. The relationship between childhood trauma and MxD was evaluated using two different multivariate models.

**Results:**

The first model (Wilks' Lambda = 0.834, *p* < 0.001) revealed that BD patients with lifetime MxD reported significantly higher CTQ total scores compared to both non‐MxD (*p* = 0.029) and HC (*p* < 0.001). When examining childhood trauma subtypes, BD patients with MxD reported significantly higher emotional abuse scores than those without MxD (*p* < 0.001). Furthermore, compared to HC, the MxD group showed significantly elevated scores in emotional abuse, emotional neglect, and physical abuse (all *p* < 0.001). The non‐MxD group scored higher than HC on physical abuse (*p* = 0.008) and physical neglect (*p* < 0.001). Regression analyses confirmed specifically emotional abuse as a significant predictor of lifetime MxD.

**Conclusions:**

The findings demonstrate a strong association between childhood trauma, particularly emotional abuse, and lifetime MxD. These results underscore the significance of childhood trauma as a key predictor of clinical outcomes in BD.

## Summary

- Significant outcomes
  ○ Individuals with bipolar disorders and lifetime mixed depression show a higher burden of childhood trauma compared with those with non‐mixed depression and healthy controls.
  ○ Emotional trauma, particularly emotional abuse, is specifically associated with mixed depressive presentations and independently predicts lifetime mixed depression.
  ○ Physical trauma represents a general vulnerability factor for bipolar depression irrespective of depressive phenotype, whereas physical neglect appears more closely related to non‐mixed depressive presentations.
- Limitations
  ○ Due to the cross‐sectional study design, we identified associations between childhood trauma and lifetime depressive phenotypes in bipolar disorders, but causality cannot be established.
  ○ Childhood trauma was assessed retrospectively using a self‐report measure, which may be influenced by recall bias and subjective reinterpretation of early experiences.
  ○ The definition of mixed depression used was limited to depressive episodes, without considering mixed features emerging in other mood states.

## 1 Introduction

The phenomenology of depressive episodes in bipolar disorders (BD) frequently encompasses features traditionally associated with hypomania or mania, such as irritability and agitation [1]. These clinical features challenge traditional diagnostic boundaries and have led to increased interest in the concept of Mixed Depression (MxD). From the strict DSM‐IV‐TR criteria for mixed state [2], which required the simultaneous presence of full manic and depressive syndromes, to the broader DSM‐5 mixed features specifier [3], the definition and classification of MxD have experienced nosological changes over time. Yet, these diagnostic frameworks often fail to capture clinically relevant dimensions of MxD, where excitation and depression co‐occur without a full syndromic overlap [4, 5, 6]. Athanasios Koukopoulos' conceptualization of MxD represents a clinically grounded response to these diagnostic limitations, extending beyond the classical manic symptomatology to include broader aspects of psychic excitation, such as inner tension and mood lability [7]. These criteria are particularly relevant in clinical practice, where MxD represents a clinically significant yet frequently underdiagnosed core presentation of BD, often following a recurrent pattern over the lifetime course of the disease [8]. Evidence suggests that in BD, MxD is more prevalent than non‐MxD and has a more complex clinical course [9, 10], including decreased response to pharmacological treatments, heightened risk of suicide, and a more pronounced familiarity with BD [5, 10, 11, 12, 13, 14, 15, 16].

Among the other environmental risk factors associated with a more severe course of BD, childhood trauma stands out as one of the most impactful [17, 18]. Defined as exposure to abuse or neglect during crucial developmental stages, childhood trauma has emerged as a major environmental element affecting the onset, course, and expression of BD [19]. Across both type I and type II subtypes, individuals with BD report markedly elevated rates of childhood trauma compared to the general population, with prevalence estimates above 50% [20]. An expanding body of research has linked early negative experiences to a more severe and complex trajectory of illness. A history of childhood trauma has been associated with an earlier onset, increased manic and depressive episode recurrence, a more rapid cycling pattern, and increased severity of mood episodes [21, 22, 23]. Moreover, childhood trauma correlates with multiple adverse clinical outcomes, including an elevated risk of suicidal behaviors and attempts, greater functional impairment, and increased psychiatric and medical comorbidities, particularly substance use disorders [21, 22, 24, 25, 26].

Although the effects of childhood trauma and MxD on the severity of illness in BD have garnered increasing attention on their own, little is known about how these two dimensions may interact. To date, no studies have specifically investigated the association between early traumatic experiences and the lifetime tendency to present with mixed features during depressive episodes in individuals with BD. Given that MxD and childhood trauma are both linked to higher clinical severity, elevated suicidality, and decreased responsiveness to treatment [8, 13, 21, 25], this gap becomes particularly noticeable.

### 1.1 Aims of the Study

The present study aims to explore the association between childhood trauma and mixed depression in a large, well‐characterized sample of individuals with both bipolar disorders type I and II, with the goal of clarifying whether early adverse experiences may be associated with mixed depressive phenomenology.

## 2 Materials and Methods

### 2.1 Participants

A total of 220 outpatients diagnosed with BD according to DSM‐5 criteria were consecutively recruited between February 2023 and July 2025 from the outpatient mood disorders clinic of the Psychiatry Department of the Fondazione Policlinico Universitario Agostino Gemelli IRCCS in Rome, Italy. Patients were approached during routine clinical visits and invited to participate in the study. All individuals who provided consent underwent a standardized eligibility screening conducted by trained psychiatrists in accordance with the study's inclusion and exclusion criteria. BD diagnosis was confirmed using the Structured Clinical Interview for DSM‐5 (SCID‐5; [27]). Inclusion criteria required participants to meet the following: (a) age between 18 and 65 years; (b) at least 5 years of formal education; (c) fluency in Italian; (d) stable pharmacological treatment for a minimum of 3 months. Exclusion criteria were: (a) a history of psychosis unrelated to the primary mood disorder; (b) traumatic brain injury with loss of consciousness; (c) major medical or neurological conditions; (d) current substance use disorder. In addition, 156 healthy controls (HC) were assessed. All HCs were screened for any current or lifetime history of DSM‐5‐TR disorders using the SCID‐I/NP [28] and SCID‐II [29]. Furthermore, a detailed family history was obtained to assess for any mood disorders or schizophrenia in first‐degree relatives. Participants with a positive family history of these conditions were excluded. The exclusion criteria applied to the HC group were identical to those used for the patient group. Ethical approval for the study was granted by the local ethics committees (protocol number: 5016, 23 January 2023) in compliance with the World Medical Association's (WMA) Declaration of Helsinki, initially adopted at the 18th General Assembly in Helsinki, Finland (June 1964) and revised at the 64th General Assembly in Fortaleza, Brazil (October 2013). All participants provided written informed consent after being fully informed about the objectives and procedures of the study. No financial compensation was provided to participants.

### 2.2 Clinical and Psychopathological Assessments

#### 2.2.1 Clinical Assessment and Lifetime Mixed Depression

A semi‐structured interview conducted by a senior psychiatrist collected anamnestic and clinical data on mood episodes, including a retrospective lifetime assessment of depressive episodes, in line with previous methodologies used in related research [30]. The interview was based on clinical evaluation and adhered to DSM‐5 diagnostic criteria. Rather than binary yes/no responses, the interview was based on flexible question formulations in order to capture nuanced clinical insights and to improve patient understanding. Final evaluations were informed by collateral information from family members or close friends and medical documentation. All data collected, including demographics, family history, psychiatric history, and clinical course variables, were systematically documented on standardized medical forms and later processed through computerized analyses.

Lifetime depressive episodes were reconstructed through detailed clinical evaluation and lifetime charts (an example of a completed lifetime chart is available in Figure S1). Patients were classified as having MxD if > 50% of their lifetime depressive episodes met the Koukopoulos' diagnostic criteria for mixed depression [7, 31]. The diagnosis of MxD according to the Koukopoulos' criteria requires the presence of a Major Depressive Episode (MDE) as defined by DSM‐5 criteria, along with at least three of the following eight features: (1) absence of psychomotor retardation; (2) talkativeness; (3) psychic agitation or inner tension; (4) subjective reports of suffering or spontaneous weeping episodes; (5) racing or crowded thoughts; (6) irritability or unprovoked outbursts of rage; (7) mood lability or marked affective reactivity; and (8) early insomnia (see Table S1).

#### 2.2.2 Childhood Trauma

The assessment of childhood traumatic experiences was conducted using the Childhood Trauma Questionnaire (CTQ) [32]. The CTQ is a retrospective, self‐administered questionnaire specifically developed to capture a broad range of maltreatment experiences occurring during the developmental years of childhood and adolescence. Its design allows for the systematic assessment of both overt forms of abuse and more subtle manifestations of neglect, thereby providing a multidimensional profile of early‐life adversities. The instrument is composed of 28 items, each formulated as a statement referring to past experiences within the family or caregiving context. Respondents are asked to rate the extent to which each statement corresponds to their personal history on a 5‐point Likert scale, ranging from “never true” to “very often true.” Of these 28 items, 25 assess five distinct trauma subtypes, specifically emotional abuse, physical abuse, sexual abuse, emotional neglect, and physical neglect, each yielding a subscale score ranging from 5 to 25. Together, these may be aggregated into a total CTQ score ranging from 25 to 125, with higher values indicating greater severity of childhood maltreatment. In addition, the CTQ includes three Minimization/Denial items, which do not contribute to the total trauma score but serve as validity indicators to identify potential underreporting or denial of traumatic experiences. The CTQ has demonstrated robust psychometric properties. It has consistently shown strong internal consistency and test–retest reliability [33]. Importantly, its validity has been established in both community‐based, non‐clinical samples [34] and in diverse clinical populations [35, 36]. The CTQ has been extensively used in cohorts with affective disorders, highlighting its utility in elucidating the role of early adverse experiences in the pathophysiology and clinical presentation of mood disorders [37, 38, 39, 40].

### 2.3 Statistical Analyses

First, we divided our sample into three groups: patients with Mixed Depression (MxD) and Non‐Mixed Depression (non‐MxD), based on the psychopathological assessments of their lifetime history of depressive episodes following the Koukopoulos' criteria [5, 41]. Second, we conducted a series of Chi‐Squared tests and one‐way ANOVA, with the groups as the independent variable and categorical measures, including sociodemographic and clinical traits, as well as the total score obtained on clinical assessments of childhood trauma (CTQ), as the dependent variables. The aim was to evaluate potential differences in these variables among groups of participants classified as MxD, non‐MxD, and the HC group. Significance was set at *α* = 0.05.

The relationship between childhood trauma and MxD was evaluated using two different multivariate models. In the first model, we investigated the distribution patterns of childhood trauma subtypes in the three groups (i.e., MxD, non‐MxD, and HC). Therefore, we conducted a multivariate analysis of variance (MANOVA) with all the subtypes of childhood trauma as dependent variables and the different groups as the independent factor. If the initial model was significant, we performed a series of one‐way ANOVAs to compare means among groups, followed by Scheffé's post hoc tests to identify specific group differences. Levene's test was used to assess the assumption of homogeneity of variances. When Levene's test was significant, Welch's correction was applied. The level of significance for the MANOVA was set at *α* < 0.05. For comparative measurements, we applied a statistical model with a Bonferroni correction (adjusted *α*: 0.05/number of comparisons = 0.01) to reduce the likelihood of Type I errors.

In the second model we specifically investigated predictors of lifetime MxD in BD. Accordingly, we conducted, only for BD patients, a series of logistic regression analyses to assess the contribution of the scores obtained in the clinical assessments of childhood trauma (CTQ), together with age, gender, and education, as predictor variables for depression type. This approach allowed us to explore the influence of each childhood trauma subtype on lifetime MxD, controlling for the combined effects of possible confounders. To account for multiple comparisons and reduce the likelihood of Type I errors, a Bonferroni correction was applied to the results (adjusted *α*: 0.05/number of comparisons = 0.012). Multicollinearity between the predictor variables was assessed [42] using tolerance [43] and VIF (variance inflation factor; [44]) values. To conclude, multivariate normality was systematically assessed as part of the analyses [45]. All statistical analyses were performed using JASP (Version 0.19.1; [46]) and SPSS (Version 29.0.1.0; [47]).

## 3 Results

In the total sample of participants (*N* = 376), the mean age was 44.13 (SD = 14.25), the mean education level was 14.49 (SD = 3.54), while 205 participants were females (54.5%) and 171 males (45.5%). Participants were allocated to three groups: 100 patients classified as MxD (45.5%) and 120 classified as non‐MxD (54.5%) according to the Koukopoulos criteria, as well as 156 healthy controls (HC). The sociodemographic and clinical characteristics of the three groups, MxD, non‐MxD, and HC, are shown in Table 1. As reported, the only significant difference among the groups concerned education level.

**TABLE 1:** Sociodemographic and clinical characteristics of the total sample of participants.

Variables	MxD *N* = 100	Non‐MxD *N* = 120	HCs *N* = 156	*F* or *χ* ^2^	df	*p*	Effect size (*η* ^2^_p_ or V)
Age (y) Mean ± SD	43.73 ± 11.52	44.44 ± 14.26	44.14 ± 15.82	0.086*	2	0.918	3.632 × 10^−4^
Gender, *N* (%)				3.110	2	0.211	0.091
Male	45.00 (45.0%)	62.00 (51.7%)	64.00 (41.0%)				
Female	55.00 (55.0%)	58.00 (48.3%)	92.00 (59.0%)				
Education, (y) Mean ± SD	14.21 ± 3.47	13.75 ± 3.77	15.24 ± 3.27	6.600	2	0.002	0.034
Married, *N* (%)				3.877	2	0.144	0.102
Yes	70.00 (70.0%)	81.00 (67.5%)	92.00 (59.0%)				
No	30.00 (30.0%)	39.00 (32.5%)	64.00 (41.0%)				
Smoking, *N* (%)				0.946	2	0.623	0.050
Yes	42.00 (42.0%)	56.00 (46.7%)	64.00 (41.0%)				
No	58.00 (58.0%)	64.00 (53.3%)	92.00 (59.0%)				
Family Psychiatric History, *N* (%)				2.015	1	0.156	0.096
Yes	70.00 (70.0%)	73.00 (60.8%)	—				
No	30.00 (30.0%)	47.00 (39.2%)					
Hospitalization, *N* (%)				2.464	1	0.116	0.106
Yes	52.00 (52.0%)	75.00 (62.5%)	—				
No	48.00 (48.0%)	45.00 (37.5%)					
Age at onset (y) Mean ± SD	29.67 ± 11.72	29.44 ± 12.43	—	0.021	1	0.884	8.936 × 10^−5^
BD type, *N* (%)			—	0.427	1	0.514	0.042
BD1	56.00 (48.28%)	56.00 (44.10%)					
BD2	60.00 (51.72%)	71.00 (55.91%)					
Seasonality, *N* (%)			—	0.064	1	0.801	0.016
Yes	33.00 (28.45%)	38.00 (29.92%)					
No	83.00 (71.55%)	89.00 (70.08%)					
Switch, *N* (%)			—	0.085	1	0.770	0.019
Yes	33.00 (28.45%)	34.00 (26.77%)					
No	83.00 (71.55%)	93.00 (73.23%)					
AD, *N* (%)				1.099	1	0.295	0.071
Yes	38.00 (38.0%)	54.00 (45.0%)	—				
No	62.00 (62.0%)	66.00 (55.0%)					
AP, *N* (%)				< 0.001	1	0.980	0.002
Yes	59.00 (59.0%)	71.00 (59.2%)	—				
No	41.00 (41.0%)	49.00 (40.8%)					
AE, *N* (%)				0.243	1	0.622	0.033
Yes	45.00 (45.0%)	58.00 (48.3%)	—				
No	55.00 (55.0%)	62.00 (51.7%)					
Li, *N* (%)				0.547	1	0.460	0.050
Yes	50.00 (50.0%)	54.00 (45.0%)	—				
No	50.00 (50.0%)	66.00 (55.0%)					

*Note: F*‐Values marked with * were computed using Welch's correction due to violation of the homogeneity of variances assumption.

Abbreviations: AD: antidepressant therapy; AE: antiepileptic therapy; AP: antipsychotic therapy; df, degrees of freedom; HC(s): healthy control(s); Li: lithium; MxD: mixed depression; *N*, number of observations; non‐MxD: non‐mixed depression; SD, standard deviation; y, years.

The three groups of participants did not differ significantly in sociodemographic or clinical characteristics (Table 1).

The ANOVA showed significant differences between all of the groups for CTQ total scores [*F*(2) = 18.875, *p* < 0.001, *ƞ*_p_^2^ = 0.091]. Post hoc analyses highlighted that both patient groups obtained higher scores than HCs [MxD vs. HCs: *p* < 0.001; non‐MxD vs. HCs: *p* = 0.004]. In addition, the MxD group exhibited higher CTQ total scores than non‐MxD [*p* = 0.029].

### 3.1 Distribution Patterns of Childhood Trauma Subtypes in the Three Groups

The preliminary MANOVA revealed a significant global effect of the groups of participants (Wilks' Lambda = 0.834, *F* = 6.992, df = 10, *p* < 0.001). Based on this result, we subsequently conducted a series of one‐way ANOVAs to compare means among groups (Table 2).

**TABLE 2:** Childhood Trauma Questionnaire assessment in the total sample of participants.

Variables	MxD	Non‐MxD	HCs	*F*	df	*p*	Effect size (*η* ^2^_p_)	Scheffé post hoc test	Scheffé post hoc test	Scheffé post hoc test
	(*N* = 100)	(*N* = 120)	(*N* = 156)					MxD vs non‐MxD	MxD vs HCs	Non‐MxD vs HCs
	Mean ± SD	Mean ± SD	Mean ± SD					*p*	*p*	*p*
Emotional abuse	9.13 ± 5.25	7.16 ± 2.86	6.35 ± 2.21	14.006*	2	< 0.001	0.097	< 0.001	< 0.001	0.156
Emotional neglect	11.94 ± 4.51	10.71 ± 4.93	9.56 ± 3.99	9.490*	2	< 0.001	0.045	0.125	< 0.001	0.105
Physical abuse	5.78 ± 1.49	5.54 ± 1.38	5.11 ± 0.41	13.991*	2	< 0.001	0.058	0.297	< 0.001	0.008
Physical neglect	6.55 ± 1.72	6.98 ± 2.21	6.12 ± 1.56	6.995*	2	0.001	0.039	0.219	0.199	< 0.001
Sexual abuse	5.56 ± 1.25	5.55 ± 1.33	5.38 ± 0.95	1.168*	2	0.313	0.006	0.998	0.476	0.478
CTQ total	38.96 ± 9.74	35.94 ± 9.07	32.52 ± 6.64	18.875*	2	< 0.001	0.091	0.029	< 0.001	0.004

*Note: F*‐Values marked with * were computed using Welch's correction due to violation of the homogeneity of variances assumption.

Abbreviations: CTQ: Childhood Trauma Questionnaire; df, degrees of freedom; HC(s), Healthy Control(s); MxD: mixed depression; *N*, number of observations; non‐MxD: non‐mixed depression; SD, standard deviation.

**FIGURE 1:**
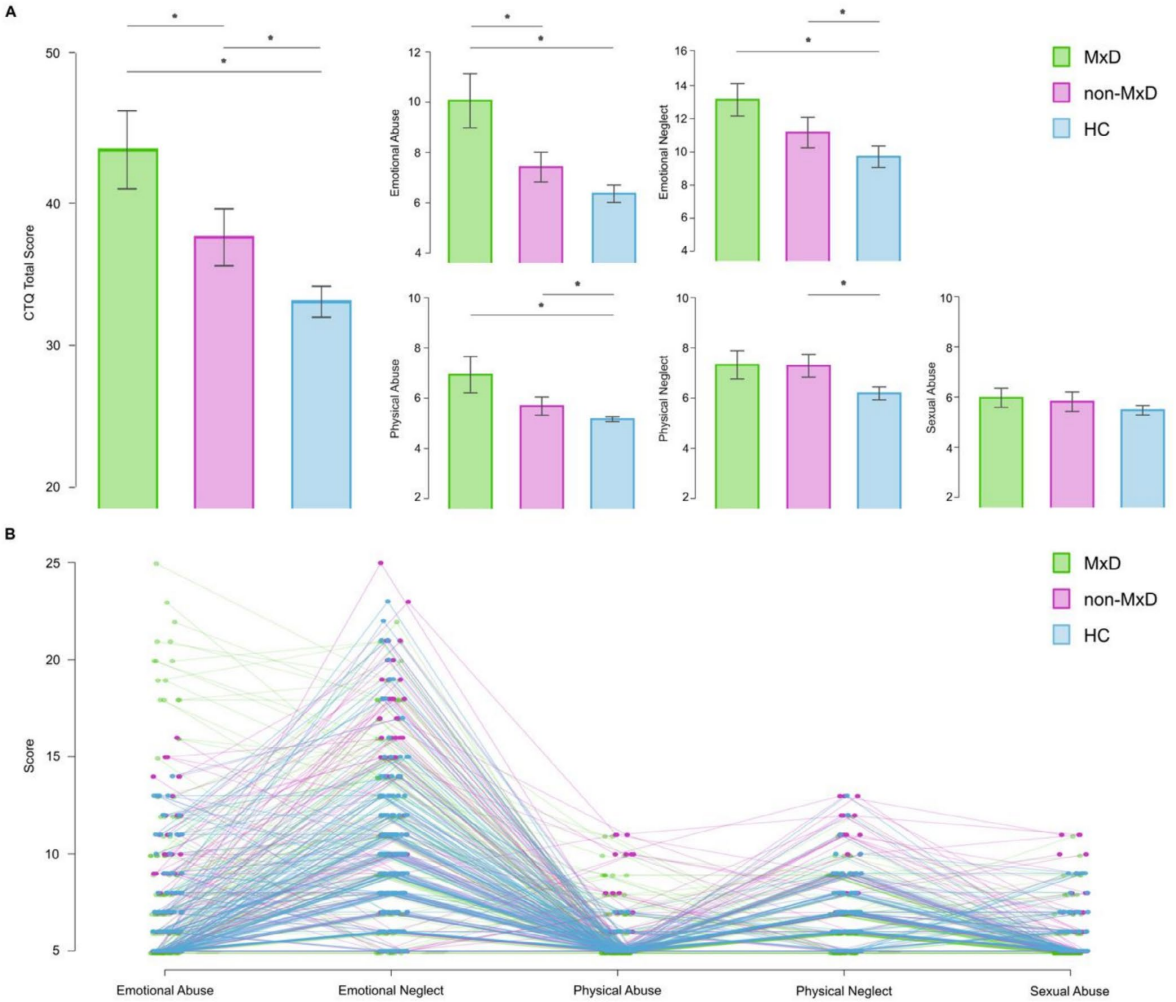
(A) Bar plots illustrating the scores obtained by mixed depression (MxD), non‐mixed depression (Non‐MxD), and healthy controls (HC) groups in the Childhood Trauma Questionnaire (CTQ). (B) Individual score distributions across all subscales and groups. Asterisks (*) indicate statistically significant group differences.

The series of one‐way ANOVAs highlighted the presence of a significant difference among groups for all the scores obtained in the subscales of the CTQ (*p* < 0.001), except for the Sexual Abuse subscale (*p* = 0.313, Figure 1). In the Emotional Abuse subscale, MxD showed a higher score compared to non‐MxD and HC (*p* < 0.001), while no significant difference emerged between non‐MxD and HC (*p* = 0.156), as indicated by the pairwise Scheffé post hoc analyses. For Emotional Neglect, MxD showed a higher score compared to HC (*p* < 0.001), while no significant difference was found compared to non‐MxD (*p* = 0.125) or between non‐MxD and HC (*p* = 0.105). Regarding Physical Abuse, the MxD (*p* < 0.001) and non‐MxD (*p* = 0.008) group obtained higher scores compared to HC, while no significant difference was found between the two groups of patients. Finally, in the Physical Neglect subscale non‐MxD obtained a higher score compared to HC (*p* < 0.001), while no significant difference was found compared to MxD (*p* = 0.219) or between MxD and HC (*p* = 0.199).

### 3.2 Predictors of Lifetime MxD in BD

Emotional Abuse was a significant predictor (Δ*Χ* ^2^ (215) = 12.540, *p* = 0.014) of MxD (Odds Ratio = 1.121, *z* = 3.066, Wald = 9.401, *p* = 0.002), suggesting that for each unit increase in the emotional abuse score, the odds of being in the MxD group increased by 12.1%. The other predictors, gender (Odds Ratio = 1.074, *z* = 0.249, Wald = 0.062, *p* = 0.803), age (Odds Ratio = 0.999, *z* = −0.059, Wald = 0.003, *p* = 0.953), and education (Odds Ratio = 1.019, *z* = 0.481, Wald = 0.232, *p* = 0.630) were not significant in predicting membership in the MxD group. The models that used the other subscales of CTQ and the CTQ total score were not significant: Emotional Neglect [Δ*Χ* ^2^ (215) = 5.585, *p* = 0.232], Physical Abuse [Δ*Χ* ^2^ (215) = 3.258, *p* = 0.516], Physical Neglect [Δ*Χ* ^2^ (215) = 3.694, *p* = 0.449], and Sexual Abuse [Δ*Χ* ^2^ (215) = 2.020, *p* = 0.732]; CTQ total [Δ*Χ* ^2^ (215) = 7.114, *p* = 0.130]. The multicollinearity analysis showed that the obtained values in this study (Gender: Tolerance = 0.937–0.982, VIF = 1.018–1.067; Age: Tolerance = 0.938–0.959, VIF = 1.042–1.066; Education: Tolerance = 0.921–0.975, VIF = 1.025–1.085; CTQ Total Score: Tolerance = 0.971, VIF = 1.030; Emotional Abuse: Tolerance = 0.937, VIF = 1.067; Emotional Neglect: Tolerance = 0.966, VIF = 1.035; Physical Abuse: Tolerance = 0.995, VIF = 1.005; Physical Neglect: Tolerance = 0.914, VIF = 1.095; Sexual Abuse: Tolerance = 0.998, VIF = 1.002) were all above the threshold of 0.1 for Tolerance, indicating low multicollinearity, and the VIF values for the independent variables were below 4, suggesting no significant collinearity issues.

The assessment of multivariate normality indicated no significant deviation from normality in the data distribution. The Mahalanobis distance was smaller than the critical value (all *p* > 0.001, as recommended by [48]), and the Mardia multivariate kurtosis index was 10.82, below the reference value of 35 [49, 50].

## 4 Discussion

This study aimed to investigate the relationship between childhood trauma subtypes and lifetime history of depressive phenotypes in BD. A significant difference emerged among CTQ total scores across all groups, with MxD and non‐MxD showing significantly higher scores compared to HCs, and MxD higher than non‐MxD. When examining childhood trauma subtypes, regarding emotional trauma, MxD reported significantly higher levels of emotional abuse compared to non‐MxD and HCs, as well as significantly higher levels of emotional neglect compared to HCs. Regarding physical trauma, both MxD and non‐MxD reported significantly higher physical abuse scores relative to HCs, while the non‐MxD group also showed significantly greater exposure to physical neglect compared to HCs. No significance emerged in sexual abuse among groups. Emotional abuse also emerged as a significant predictor of lifetime MxD.

Based on the differences in CTQ total scores between groups, our results revealed a clear gradient of trauma exposure, with childhood trauma severity increasing progressively from controls to BD with non‐mixed depression to BD with mixed depression (Figure 1). This pattern is consistent with extensive literature showing that individuals with BD are more likely to experience early‐life trauma than the general population [25, 51]. Our findings contribute to this body of research by suggesting that childhood trauma is not only associated with BD in general but may be specifically linked to depressive episodes with mixed features, potentially indicating a relationship between early adversity and a more activated, high‐energy depressive presentation. Some previous studies support this interpretation. A systematic review conducted by our group [52] found a possible indirect correlation between the experience of childhood trauma and the development of mood episodes with mixed features. Similarly, an early study found a correlation between childhood trauma and current mixed episodes in patients with BD type I and comorbid substance use disorders [53]. Compared with our study, however, that investigation focused on a different population, did not perform a lifetime assessment of mixed depression, and did not apply Koukopoulos' criteria. Conversely, another study examining the relationship between childhood trauma and clinical features in patients with BD‐I found no association with mixed episodes [54]. Notably, in contrast to our methodology, their investigation relied on a clinical data form assessing the lifetime occurrence of at least one mixed episode and utilized the Childhood Abuse and Neglect Questionnaire (CANQ) rather than the CTQ. In addition, their sample size was relatively small. Interestingly, a study by Aas et al. [55] identified affective lability as a possible mediator in the relationship between childhood trauma and both mixed depression and suicidality. This observation is grounded in evidence that depressive episodes with mixed features correlate with a greater risk of suicidal ideation compared to inhibited forms of depression [56], and that both mixed states and childhood trauma independently increase the risk of suicide attempts [57]. Collectively, our findings, integrated with prior literature, corroborate the hypothesis that high‐energy depressive states within BD, such as those characterized by mixed features, are more likely to be associated with higher levels of childhood trauma.

To further clarify this association, we examined childhood trauma subtypes separately. These analyses identified specific distribution patterns that differentially associate emotional and physical trauma with mixed and non‐mixed depression. Emotional trauma, in both its abuse and neglect forms, resulted to be more closely associated with mixed depressive presentations than with non‐mixed forms. This observation suggests that early adversity impacting emotional development may be associated with the pathophysiology of high‐energy depressed episodes. Consistent with this finding, a meta‐analysis by Palmier‐Claus et al. [58] reported that individuals with BD are more than twice as likely to have experienced trauma during childhood than HC, with emotional abuse emerging as the subtype of trauma most strongly associated with BD. Another study confirmed this latter result and found emotional abuse not only to be the most significant but also the only one linked to BD with a dose–response relationship [40]. Our findings expand this body of literature by demonstrating that the subtype of emotional trauma is not only associated with BD broadly but with a specific depressive phenotype (i.e., mixed depression). This may be partially addressed by the well‐documented association between childhood emotional trauma and impaired neural circuits involved in affective modulation. As a consequence, emotional abuse and neglect may predispose individuals to increased emotional reactivity and reduced affective control in adulthood [59, 60]. These patterns of emotional dysregulation closely mirror the affective core of mixed depression, where affective lability and hyperreactivity have been identified among the diagnostic criteria [7]. This hypothesis is supported by previous studies identifying a specific relationship between affective lability and childhood trauma exposure [61, 62], with emotional abuse being the most strongly associated [55, 63]. However, previous studies have also documented hypersensitivity to emotional stimuli in patients with BD, even during euthymic phases [64, 65]. This raises the question of whether childhood emotional trauma directly fosters affective lability and thereby increases vulnerability to mixed states, or whether the intrinsic emotional hypersensitivity of BD amplifies the subjective impact of early interpersonal adversity [62]. Future longitudinal research will be essential to clarify whether early trauma represents a causal antecedent or rather a reinforcing correlate of a pre‐existing vulnerability.

Another interesting result emerging from our results is the significance of physical trauma as a broader vulnerability factor in BD, regardless of depressive phenotype. According to earlier research, childhood physical abuse is more common among BD than in the general population, which emphasizes the possible influence of this trauma subtype on the onset and the clinical presentation of the illness [66]. In fact, physical trauma has been associated with a more severe illness trajectory in BD, such as earlier age at onset, greater episode recurrence, higher comorbidity with anxiety and substance use disorders, and poorer psychosocial performances [19]. Large‐scale epidemiological data also support these findings. Physical abuse during childhood was linked to a 41% higher risk of mood disorders, including BD, according to the National Epidemiologic Survey on Alcohol and Related Conditions (NESARC) [67]. Such findings converge with recognized etiological models, including the diathesis‐stress paradigm, which claims that environmental factors such as physical trauma interact with latent vulnerabilities to trigger illness onset [68, 69, 70]. In our sample, the lack of differentiation among depressive subtypes supports the notion that physical abuse is broadly associated with bipolar depression, regardless of energy levels or specific phenotypic presentation. Furthermore, the absence of differences in physical abuse between the two depressive phenotypes may also reflect the high prevalence of family history in both subgroups. Given that BD has been linked to heightened irritability, impulsive behavior, and unstable interpersonal dynamics within affected families, such shared environmental conditions could contribute to the uniform distribution of physical abuse across depressive phenotypes [38, 71]. Unlike physical abuse, which appeared to act as a general vulnerability factor across depressive phenotypes, physical neglect emerged as significantly related only to non‐MxD. This pattern suggests that physical neglect may represent a clinical marker of more inhibited or melancholic depressive states, which are typically characterized by psychomotor retardation, somatic involvement, and neurovegetative disturbances, pointing to a possible link between early trauma and bodily domains of affective dysregulation [72, 73]. This interpretation resonates with the conceptualization of melancholic or non‐mixed depression as a “blocked” form of depression, in which somatic constriction and psychomotor inhibition predominate [74]. It is conceivable that prolonged exposure to neglect could disrupt regulatory circuits implicated in both stress responsivity and energy balance, predisposing individuals to more inhibited rather than agitated mood states [75].

From a clinical perspective, these findings also hold implications for treatment approaches for trauma‐exposed individuals with BD, particularly those presenting with mixed depressive features. Trauma‐focused psychotherapies, including EMDR and trauma‐focused CBT, have shown promising results in this subgroup, with preliminary studies demonstrating reductions in depressive symptomatology and improvements in overall mood stability, including mixed features [76, 77]. On the pharmacological side, mood stabilizers and atypical antipsychotics remain central in the management of bipolar depression, including mixed depressive states, given their established antidepressant properties and relapse‐prevention effects [78, 79]. These findings support the notion that incorporating trauma‐informed frameworks may enhance the management of depressive states in BD, particularly when mixed features are prominent.

### 4.1 Limitations

Some limitations must be acknowledged before presenting the conclusions of this study. The cross‐sectional design provides a precise overview of the potential relationship between childhood trauma and lifetime depressive phenotypes in bipolar depression. However, since data were collected at a single time point, this design does not allow to determine the direction of the possible cause‐effect relationships. Nevertheless, the large sample size and the application of detailed clinical evaluation and lifetime charts to assess the lifetime history of mixed depression reinforce the validity of the results and set the basis for future longitudinal studies. Furthermore, the self‐report nature of the Childhood Trauma Questionnaire (CTQ) may be subject to recall bias and self‐perception distortions. However, this potential bias is likely similar across all groups and therefore does not affect between‐group comparisons but rather represents a general limitation of retrospective assessments. Nonetheless, to date, the CTQ remains one of the most widely used and validated tools for retrospective evaluation of early life trauma [32]. Moreover, the definition of mixed depression we used does not match the DSM‐IV criteria for mixed depression nor the mixed depression specifier of the DSM‐5. This makes our study not comparable to former research using the DSM‐IV and some of the more recent DSM‐5‐based studies. Furthermore, since Koukopoulos' criteria assess mixed features exclusively within depressive episodes, the presence of mixed features during manic or hypomanic episodes was not evaluated. However, the instrument was specifically chosen because it allows for a more nuanced characterization of mixed depression, enabling its application in real‐world clinical practice for BD and addressing the limitations and criticisms of DSM‐based diagnostic criteria [4, 9].

### 4.2 Conclusions

In conclusion, the present study presents new evidence that childhood trauma is differentially associated with lifetime depressive subtypes in BD. Emotional trauma appears to be more specifically associated with mixed depressive presentations, likely due to its lasting impact on affective regulation, whereas physical abuse represents a broader, non‐specific risk factor linked to bipolar depression in general. Since mixed depression represents a more severe and clinically challenging form of mood disorder, often associated with treatment resistance and elevated suicide risk [12, 13, 15], clarifying the early‐life factors associated could facilitate diagnosis and guide treatment strategies. Therefore, these findings support the importance of systematic assessment of childhood trauma and lifetime characteristics of depression in BD. Future research should focus on investigating causal pathways through longitudinal designs, investigating biological mediators of this relationship, and assessing whether addressing emotional dysregulation in trauma‐exposed individuals can impact mixed depressive features in individuals with BD.

## Author Contributions

Conceptualization: Francesca Bardi and Delfina Janiri. Data curation: Mario Pinto, Alessio Simonetti, and Marco Di Nicola. Formal analysis: Mario Pinto and Francesca Bardi. Investigation: Marianna Mazza and Giovanni Manfredi. Methodology: Francesca Bardi and Georgios D. Kotzalidis. Supervision: Gabriele Sani and Delfina Janiri. Validation: Gabriele Sani, Delfina Janiri. Visualization: Francesca Bardi, Mario Pinto. Writing – original draft: Francesca Bardi and Mario Pinto. Writing – review and editing: Francesca Bardi and Delfina Janiri.

## Funding

The authors have nothing to report.

## Ethics Statement

The study was conducted in accordance with the Declaration of Helsinki and approved by the Ethics Committee of Fondazione Policlinico Agostino Gemelli IRCCS (protocol number: 5016; date of approval: 23 January 2023).

## Consent

Written informed consent was obtained from all participants involved in the study.

## Conflicts of Interest

The authors declare no conflicts of interest.

## Supporting information


**Data S1:** acps70071‐sup‐0001‐supinfo.docx.

## Data Availability

The data that support the findings of this study are available on request from the corresponding author. The data are not publicly available due to privacy or ethical restrictions.
