# Supplementary material for: Lifetime Mixed Depression and Childhood Trauma in Individuals With Bipolar Disorders
Source: Acta Psychiatr Scand. 2026 Jan 25;153(4):290–300. doi: 10.1111/acps.70071 (PMC12967745; doi:10.1111/acps.70071)
Supplement: Supplementary file 1 — Data S1: acps70071‐sup‐0001‐supinfo.docx. [file ACPS-153-290-s001.docx]

**Supplementary material**

**Supplementary Table S1**. Koukopoulos' diagnostic criteria for mixed depression.

| **Major depressive episode + at least 3 of 8 items** |
| --- |
| - Psychic agitation or inner tension - Racing or crowded thoughts - Irritability or unprovoked rage - Absence of retardation - Talkativeness - Dramatic description of suffering or frequent spells of weeping - Mood lability or marked reactivity - Early insomnia |

**Supplementary Figure S1**. Example of life chart used to evaluate lifetime depressive episodes.
